# Noninterventional study assessing joint health in persons with hemophilia A after switching to turoctocog alfa pegol: design of pathfinderReal

**DOI:** 10.1016/j.rpth.2024.102363

**Published:** 2024-03-01

**Authors:** Cihan Ay, Olga Benitez-Hidalgo, Gillian Gidley, Maria Elisa Mancuso, Davide Matino, Azusa Nagao, Laszlo Nemes, John Waller, Johannes Oldenburg

**Affiliations:** 1 Clinical Division of Haematology and Haemostaseology, Department of Medicine I, Medical University of Vienna, Vienna, Austria; 2 Hemophilia Unit, Hematology Department, Hospital Vall d'Hebron, Barcelona, Spain; 3 Haemophilia Comprehensive Care Centre, Department of Haematology, Leeds Teaching Hospitals Trust, Leeds, United Kingdom; 4 Centre for Thrombosis and Hemorrhagic Diseases, IRCCS Humanitas Research Hospital, Rozzano, Milan, Italy; 5 Humanitas University, Pieve Emanuele, Milan, Italy; 6 Department of Medicine, Thrombosis and Atherosclerosis Research Institute, McMaster University, Hamilton, Ontario, Canada; 7 Department of Blood Coagulation, Ogikubo Hospital, Tokyo, Japan; 8 National Hemophilia Center and Hemostasis Department, Central Hospital of Northern Pest – Military Hospital, Budapest, Hungary; 9 Novo Nordisk, Zurich, Switzerland; 10 Department of Molecular Haemostasis, University of Bonn, Bonn, Germany

**Keywords:** factor VIII, hemarthrosis, hemophilia A, joint, N8-GP

## Abstract

**Background:**

Joint damage affects the quality of life of persons with hemophilia A. The long-term safety and efficacy of turoctocog alfa pegol (N8-GP) prophylaxis in persons with hemophilia A has been investigated in pivotal phase 3 trials in children, adolescents, and adults (pathfinder program). However, there is a lack of data on joint health in adult persons with hemophilia A treated with N8-GP.

**Objectives:**

To describe the design of the ongoing pathfinderReal study investigating the joint health status in adult persons with hemophilia A after switching to N8-GP.

**Methods:**

pathfinderReal is a multicountry, noninterventional, single-arm study (NCT05621746) of joint health in adult (≥18 years) male persons with hemophilia A who have switched to N8-GP. Patients enrolled in other interventional studies and those who have previously terminated N8-GP treatment will be excluded. Approximately 124 adults with hemophilia A will be enrolled and followed up for a maximum of 24 months. Data from routine clinical assessments of patients’ joint health will be collected. The primary endpoint is change in Hemophilia Joint Health Score (defined as a change in total score of ≤2) from initiation of N8-GP treatment until the end of the study. Secondary endpoints include number of bleeding episodes, number and resolution of target joints, patient-reported outcomes of problem joint score, pain score, and change in physical function levels. An exploratory endpoint is included to measure the number of patients achieving improved Hemophilia Joint Health Score from the initiation of N8-GP until the end of the study.

**Conclusion:**

The pathfinderReal study will provide insights regarding the impact of N8-GP on joint health in persons with hemophilia A in a real-world setting.

## Essentials

• Joint damage is an important outcome affecting the quality of life of persons with hemophilia.
• Data on joint health in adult persons with hemophilia A treated with N8-GP are lacking.
• We present the design of pathfinderReal, an ongoing study evaluating joint health in adult persons with hemophilia A.
• pathfinderReal aims to investigate outcomes of N8-GP treatment on joint health in persons with hemophilia A in a real-world setting.

## 1 Introduction

Clinical outcome measures for hemophilia A (HA) include several laboratory and clinical evaluations such as factor VIII (FVIII) levels, FVIII inhibitors, overall bleeding rates, joint bleeding rates, target joints, and quality-of-life (QoL) measures [1,2]. Of all the outcome measures, joint damage largely affects QoL in persons with HA as well as influences the related socioeconomic burden of treatment, including treatment costs and work productivity [[3], [4], [5]]. Recurrent bleeding episodes into the joints may trigger synovitis, leading to progressive osteochondral damage [6] and ultimately resulting in chronic hemophilic arthropathy, which is usually characterized by chronic pain and poor physical function [6,7]. Those joints where at least 3 bleeds occur over a 6-month period are termed target joints [8]. On average, the most frequently involved joints are elbows, knees, and ankles. The functional impact of hemophilic arthropathy can be quantified using the Hemophilia Joint Health Score (HJHS) version 2.1, a validated clinical assessment tool [9]. The HJHS was originally developed to assess joint health in children with hemophilia in order to detect early signs of joint damage and has recently been validated for use in an adult population [10]. HJHS evaluates the status of a joint, including swelling, duration of swelling, muscle atrophy, crepitus of motion, range of motion (extension and flexion loss), joint pain, strength, and gait [[9], [10], [11]].

The current standard of care for HA is long-term prophylaxis, which consists of regular intravenous injections of clotting factor concentrates (either standard half-life or extended half-life products) or regular subcutaneous injections of FVIII mimetics, including episodic treatment in case of breakthrough bleeds [12,13]. In persons with HA, regular prophylaxis with FVIII products is effective in preventing recurrent bleeding events in muscles and joints [13,14]. Turoctocog alfa pegol (N8-GP) is an extended half-life, recombinant, glycoPEGylated FVIII product used for both the prevention and treatment of bleeding episodes in persons with HA [15]. N8-GP offers convenient prophylaxis treatment with a lower frequency of injections (every fourth day) than standard half-life products (on average, every second day or 3 times/wk), with mean trough levels of 3.0 IU/dL and 2.7 IU/dL in adults and adolescents, respectively [16]. This provides a 1.6 times longer half-life in adults compared with standard FVIII products [17]. The long-term safety and efficacy of N8-GP prophylaxis in persons with HA have been investigated in pivotal phase 3 pathfinder clinical trials in children, adolescents, and adults [17,18]. However, there is a scarcity of data regarding joint health in adults treated with N8-GP.

This manuscript outlines the design of the ongoing pathfinderReal study, which is investigating the joint health status in adult persons with HA after switching to N8-GP prophylaxis. The study will assess joint-related clinical outcomes in the patient population, including target joints, number of bleeding episodes, problem joint scores, and pain scores. The study will also evaluate patient-reported outcome (PRO) questionnaires on physical function and activity levels.

## 2 Methods

### 2.1 Study design

pathfinderReal (NCT05621746) is an international, noninterventional, single-arm study assessing the joint health of adult male persons with HA who have switched to treatment with N8-GP prophylaxis. Patients will be treated with commercially available N8-GP according to the approved local label and clinical practice at the treating physician’s discretion. Patients will be eligible to participate in the study if they have switched to N8-GP in the 18 months preceding enrollment or have decided to switch during the 2 months post enrollment. The decision to initiate treatment with N8-GP is made by the patient and treating physician prior to enrollment in the study. The total study duration for each participant is estimated to be 24 months (Figure). The observation period will be a maximum of 24 months post the switch to N8-GP, during which patient data will be collected; this will include collecting retrospective data for some patients. Independent of routine visits, the study design includes biannual remote direct data capture by participants for PROs (Figure). The study started on November 23, 2022, with the first patient enrollment, and recruitment is ongoing, with each patient observed for a maximum of 24 months.

**Figure:**
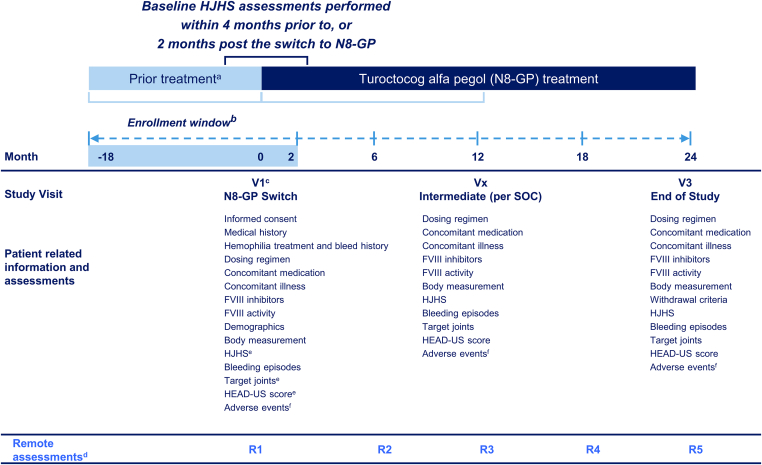
pathfinderReal study design. ^a^Prior treatment: any hemophilia treatment regime other than N8-GP received prior to study enrollment. ^b^The enrollment window: the date when the patient switched to N8-GP (within 18 months prior to enrollment) or planned to switch (within 2 months post enrollment) to prophylaxis with N8-GP from previous therapy. ^c^Visit 1 may occur prior to enrollment due to criteria that allow patients who have switched to N8-GP in the 18 months prior to enrollment. ^d^Remote PRO measurements: biannual remote direct data capture by participants is planned for the PRO assessments. ^e^Assessments performed within 4 months prior to switch or 2 months after the switch to N8-GP can be used as baseline data. ^f^Sites should ask and report on adverse events at each contact with the patient during the course of the study. FVIII, factor VIII; HEAD-US, Hemophilia Early Arthropathy Detection with Ultrasound; HJHS, Hemophilia Joint Health Score; N8-GP, turoctocog alfa pegol; PRO, patient-reported outcome; Rx, remote assessment number; SOC, standard of care; Vx, visit number.

### 2.2 Study population and eligibility criteria

Overall, patients from 30 to 40 sites across 11 countries (Austria, Canada, Germany, Italy, Denmark, Japan, Saudi Arabia, Spain, Hungary, the United Kingdom, and the United States) are included in the study. The sites were selected based on their adherence to the treatment guidelines on joint health assessments and prescription of N8-GP.

The inclusion criteria were chosen to focus the study on a clinically relevant patient population with HA. The inclusion criteria are as follows: (a) adult males ≥18 years of age at the time of initiating N8-GP who have been diagnosed with severe (FVIII activity, <1%) or moderate (FVIII activity, 1%-5%) HA; (b) those who signed informed consent obtained before study enrollment; (c) those who switched to treatment with N8-GP within 18 months prior to enrollment or planned to switch within 2 months post enrollment, independent of the decision to enroll in the study; and (d) participants with baseline data (HJHS, target joints, and medical history) collected in routine clinical practice within 4 months before or up to 2 months after switching to N8-GP treatment. The key exclusion criteria are as follows: (a) previous participation in this study; (b) history of terminated treatment with N8-GP, either on demand or for prophylaxis; (c) mental incapacity, unwillingness, or language barriers precluding adequate understanding or co-operation; and (d) current participation in interventional clinical trials.

### 2.3 Study objectives and endpoints

The primary objective of the pathfinderReal study is to evaluate if joint health is maintained in persons with HA after switching to N8-GP. The primary endpoint is to assess the change in HJHS version 2.1 (change in total score of ≤2) from the initiation of N8-GP until the end of the study. The overall score from HJHS evaluates 6 index joints and provides users with a relative indicator of joint health, with a lower HJHS representing better joint health [19]. The scoring range of the HJHS is from 0 (normal, healthy joints) to 124 (maximum severity) [9,11,20,21]. In this study, maintained HJHS is defined as a change in total score of ≤2 points in the 24 months post initiation of N8-GP. This value was determined via a consensus agreement following the review of 11 published studies, which assessed the change in HJHS over time [[22], [23], [24], [25], [26], [27], [28], [29], [30], [31], [32]]. The 11 studies, consisting of a total of 537 persons with hemophilia (the majority with severe hemophilia and on prophylaxis), reported an average change in HJHS of +2.

The secondary objective is to assess additional clinically relevant parameters associated with joint health in terms of bleeding patterns, pain, and QoL outcomes. This includes assessing target joints evaluated by the number of cases and by patient-reported problem joints, which are defined as joints having chronic pain and/or limited range of movement due to compromised joint integrity (ie, chronic synovitis and/or hemophilic arthropathy), with or without persistent bleeding [33]. Problem joints are captured as total number (count) and the number per joint (count per location) [33]. In addition, Hemophilia Early Arthropathy Detection with Ultrasound (HEAD-US) scores will be assessed. Secondary endpoints include the following: (a) number of bleeding episodes requiring FVIII treatment from date of switch to N8-GP until the end of the study; (b) number of target joints developed from date of switch to N8-GP until the end of study; (c) number of target joints resolved from date of switch to N8-GP until the end of the study; and (d) change in HEAD-US score calculated using postbaseline measurements of HEAD-US (score unit), up to and including the end of study minus baseline HEAD-US (score unit). In addition, the results from the PRO questionnaires will be evaluated as follows: (a) change in patient-reported problem joint score; (b) change in patient-reported pain scores, as measured by the Brief Pain Inventory Short Form [34,35]; and (c) change in physical function levels and activity, as measured using the 36-Item Short Form Health Survey version 2, from date of switch to N8-GP until the end of the study [34].

An exploratory endpoint is to assess the number of patients with an improved HJHS by the end of the study. An improved HJHS is defined herein as a reduction in total HJHS of >2 points in the 24 months post initiation of N8-GP. There will likely be a lag period before nontransient effects on HJHS are seen after the treatment switch to N8-GP. Therefore, any scores collected within 6 months post switch can be considered unaffected by the new treatment.

### 2.4 Baseline measurements and study visits

Baseline data are defined as the data collected at the time when a patient switched to N8-GP from their current treatment. This may require retrospective data collection for patients who switched to N8-GP within up to 18 months prior to enrollment. Available data in medical records will be entered in the electronic case report form (eCRF). Data to be collected at baseline are as follows: (a) age (in years); (b) body mass index; (c) number of target joints at the time of switch to N8-GP and details of medical history of joint health, including hemophilic arthropathy of shoulders, elbows, hips, knees, or ankles; and (d) joint replacements in knees or ankles, ankle arthrodesis, and any other hemophilic arthropathy or procedure, as available. Patients will visit the sites according to standard of care frequency. The number of visits and available data may differ between sites, but ideally, patients will visit the clinic at least once per year.

Relevant data regarding patient assessments (Figure) will be entered in the eCRF. For certain assessments (HJHS, HEAD-US, and target joints), data recorded in standard clinical practice 4 months before switch or 2 months after the switch to N8-GP will be considered baseline data. Any visit(s) in the period following the baseline visit until the end of study visit will be categorized as intermediate visits. Additionally, patients will be asked on a biannual basis to enter applicable information themselves into an electronic PRO system. Patients will also be offered the option to complete PROs via paper copies, where appropriate. Patients are allowed to withdraw from the study at any time during the study period. In such circumstances, the physician should collect any outstanding data and document the reason for discontinuation in the eCRF.

### 2.5 Study size and analysis set

The study sample size is based on a 5% level of significance and a power of 80%, with an assumed SD of 7 and expected mean change after 24 months in HJHS of 0. The primary analysis is planned to include 99 patients to assess the primary endpoint. A proportion of 20% is accounted for patient discontinuation, and therefore, we aim to enroll 124 patients to achieve a sample size of 99 patients completing the study. Descriptions and analysis of effectiveness will be based on the full analysis set, as defined in International Council for Harmonisation of Technical Requirements for Pharmaceuticals for Human Use (ICH) E9 guidelines [36]. The full analysis set includes all eligible patients based on inclusion and exclusion criteria, and the safety analysis set includes all patients exposed to N8-GP. Patients who initiate immune tolerance induction treatment will be considered withdrawals for statistical analyses, and any data collected during the immune tolerance induction treatment period will be summarized and reported separately.

### 2.6 Data management and reporting of adverse events

Data management will be assigned to the contract research organization (CRO) and overseen by Novo Nordisk. All information from this study will be captured in an electronic database maintained by the CRO under the supervision of Novo Nordisk and in accordance with country-specific laws. During the study, monitoring will be performed to ensure that the patient has adhered to planned procedures. Monitoring will be performed by a CRO according to the standards set out in Novo Nordisk’s standard operating procedure for a noninterventional study. Relevant data regarding the assessments must be entered in the eCRF, if available.

Collection and reporting of all adverse events to the CRO will be performed in a timely manner from study start to study end dates. Mandatory reporting is required in the event of an overdose, abuse, medication error, or lack of therapeutic effect, with or without an accompanying adverse reaction. The treating physician should report any adverse events (serious and nonserious) to the CRO within 3 calendar days of the event. Bleeding episodes are not considered adverse or serious adverse events unless bleeding is fatal. All serious adverse events and adverse reactions (serious and nonserious) will be followed up until the outcome of the event or reaction is “recovered” or “recovered with sequelae” and queries have been resolved. Cases will be closed with an outcome of “recovering” when the patient has completed the study and is expected by the physician to recover.

### 2.7 Statistical analysis

The difference in HJHS of patients between study start and end dates will be analyzed by analysis of covariance. For the primary endpoint, the aim is to show that the HJHS does not increase by more than 2 points after 24 months, a cutoff chosen based on existing literature [28,[37], [38], [39]].

Results from statistical analyses will be accompanied by 2-sided 95% CIs and corresponding *P* values. Categorical data will be summarized by frequency tables, while continuous data will be summarized by mean, SD, median, and minimum and maximum values. Additionally, to investigate the sensitivity of the results of the primary analysis of the primary endpoint with regard to the handling of missing data, a mixed model for repeated measurements with an unstructured covariance structure will be applied. The mixed model for repeated measurements will include number of target joints at baseline and study visits as a fixed factor and baseline age, baseline body mass index, and baseline HJHS as covariates.

### 2.8 Patient and public involvement

Patients and/or the public were not involved in the design, conduct, reporting, or dissemination plans of this research.

### 2.9 Ethics and dissemination

The study is approved by the Institutional Review Board or Independent Ethics Committee from all the participating countries. The study will be conducted in compliance with the Declaration of Helsinki and Good Pharmacoepidemiology Practice [36], Good Pharmacovigilance Practice Module VI guidelines [40], and Ethical Guidelines for Medical and Health Research Involving Human Subjects [41]. All participants will sign written informed consent and are assigned a unique identification number to maintain confidentiality.

## 3 Discussion

The pathfinderReal study will evaluate the joint health status of adult persons with HA after switching to treatment with N8-GP. To capture real-world data, an extended study enrollment window will allow patients who have switched to N8-GP in the 18 months prior to enrollment or have decided to switch in the 2 months post enrollment to participate. Every patient in the study is planned to visit for routine clinical and biannual patient-reported evaluation over a duration of 24 months. During these visits, the status of joint health will be evaluated based on HJHS and other joint-related clinical outcomes, including target joints, pain, physical function, and bleeding rates.

Encouraging target joint resolution data were collected in the phase 3 pathfinder8 study, which investigated the long-term safety and efficacy of N8-GP in patients of all ages with severe HA. In pathfinder8, a total of 160 patients were enrolled from pathfinder2 and pathfinder5 and were followed up for 104 weeks. A total of 5 patients reported 7 target joints at baseline. By the end of the study, out of these 7 target joints, 3 patients had ≥1 baseline target joint resolved, and of these 3 patients, 2 had all baseline target joints resolved [18]. This suggests that there was a beneficial effect of N8-GP in a small subgroup of patients, indicating the importance of conducting a follow-up study with a larger cohort of patients.

Joint damage is a major factor in the socioeconomic burden of hemophilia treatment, influencing treatment costs and health-related QoL (HRQoL) [[3], [4], [5]]. Approximately 90% of people with severe hemophilia suffer from joint disease, which most commonly affects elbows, knees, and ankles [42]. Data from the Cost of Hemophilia across Europe – a Socioeconomic Survey study in persons with severe HA showed that the presence of chronic synovitis had a significant negative impact on HRQoL [43]. A European study on persons with severe HA or hemophilia B with inhibitors reported an increased disease burden due to orthopedic complications, with reduced patient mobility affecting overall QoL [44]. Therefore, the pathfinderReal study aims to supplement the efficacy results of N8-GP by further evaluating joint health and its impact on HRQoL by assessing various PRO measures in persons with HA.

Some of the limitations of the study include the following: (a) retrospective initiators included in the study will be successful initiators, thereby introducing bias. However, this is unavoidable due to the rarity of hemophilia; (b) the noninterventional study design involves recording data in routine practice, as opposed to mandatory clinical assessments at prespecified time points, and may impact the quantity and quality of data collected and their subsequent interpretation; (c) there is likely to be interpatient and intersite variability in the reporting of bleeding episodes requiring FVIII treatment; and (d) missing PRO data for some patients due to the inclusion of patients who have switched to N8-GP within up to 18 months prior to enrollment may limit interpretation.

## 4 Conclusion

The pathfinderReal study will report the impact of N8-GP prophylaxis on joint health in adults with HA using real-world data. On completion, these data will guide us on the significance of routine clinical evaluation of joint health parameters with the aim of improving clinical practice and well-being of patients treated with N8-GP.
